# A qualitative investigation exploring why dance festivals are risky environments for drug use and potential adverse outcomes

**DOI:** 10.1186/s12954-022-00598-5

**Published:** 2022-02-05

**Authors:** Joseph J. Palamar, İbrahim Sönmez

**Affiliations:** 1 Department of Population Health, New York University Grossman School of Medicine, 180 Madison Avenue, Room 1752, New York, NY 10016 USA; 2 Department of Political and Social Sciences, Universitat Pompeu Fabra, Barcelona, Spain

**Keywords:** Nightlife, Dance festivals, Ecstasy, Environmental risk factors

## Abstract

**Background:**

Dance festivals have been shown to be high-risk events for use of drugs such as ecstasy/MDMA and possible adverse effects associated with use. However, few studies have examined what makes festivals such risky environments. We aimed to determine festival-specific risk factors for adverse outcomes related to drug use.

**Methods:**

In-depth interviews were conducted with 35 key informants in North America who deemed themselves experts in new psychoactive substances, and identified as drug checkers, sellers, or experienced users. Interviews were coded in an inductive manner, and we conducted thematic analysis to identify relevant themes.

**Results:**

We identified four main themes focusing on festival attendance as a risk factor for risky drug use and related outcomes: attendees inexperienced with electronic dance music parties and party drugs, risky drug purchasing, risky drug use practices, and festival-specific environmental risk factors. Festivals attract a wide array of people not experienced with party drugs, yet drugs like ecstasy are commonly sought by such individuals inside festivals. Relying on strangers inside to purchase drugs is a risk factor for purchasing adulterated product. Fear of security/police at festivals leads to risky drug-taking such as ingesting one’s full batch of drugs at the entrance. These risks are compounded by environmental factors including crowding, hot temperature, and lack of water (which lead to dehydration), long/consecutive event days (which can lead to exhaustion), and inadequate medical emergency response.

**Conclusions:**

We determined modifiable risk factors which can both inform future research and future prevention and harm reduction efforts in this scene.

## Background

In recent years, dance festivals featuring electronic dance music (EDM) have increased in popularity in the USA and throughout much of the world [1]. Beginning in the late 1980s, all-night illegal EDM dance parties called raves became popular [2, 3]. These large underground parties, which originated in the UK, were fueled by techno music and drugs such as MDMA/ecstasy. They first took place in large outdoor settings such as fields, but over time these parties tended to move indoors to illegal warehouses or legal nightclub venues. Rave culture was exported to the USA and to many other counties in the late 1980s, but throughout the 1990s, rave culture appeared to become largely limited to nightclub settings. In the early 2000s, the rave element of some of these scenes would morph into large, outdoor dance festivals. Indeed, parties at nightclubs remain popular, but the new phenomenon of dance festivals has also become popular throughout the USA and worldwide. Festivals are similar to raves, but are mainstream, legal, regulated, and often held openly on government property. Festivals are live music events (featuring DJs), and they are multiple days in duration (e.g., all day Friday through Sunday) with some being continuous in duration (with camping). Tens or hundreds of thousands of people attend each festival [4, 5]. While festivals do often attract underground ravers, a much wider, general audience attends.

Dance festivals, however, can be considered high-risk events as use of party drugs such as ecstasy/MDMA tends to be highly prevalent [6], drug-related adverse effects are common [7], and because clusters of drug-related emergencies sometimes occur [4, 8, 9]. Given the growing popularity of festivals, it is important to examine potential causes of drug-related adverse effects in order to better inform prevention and harm reduction efforts.

Illegal drug use has been found to be particularly prevalent among festival attendees [6, 10], with prevalence of use higher than use among the general population [11]. For example, in an Australian study, 73.4% of attendees of a major music festival reported use of illegal drugs in the past 12 months, mostly ecstasy (59.8%) [12]. Further, individuals who have never used party drugs often initiate use at dance festivals. In a study of 1,020 EDM event-attending adults, 33.4% reported initiation of use of ecstasy at an EDM festival [13].

Festivals, however, are not only risky because high levels of drug use occur, but they also appear to be particularly risky contexts for drug-related adverse effects due to extreme environmental conditions. Festivals are usually multiple days in duration in which attendees can be exposed to heat and crowding, and over-exertion through dancing can lead to dehydration and exhaustion, which presents a unique risk for medical emergencies such as heat stroke, particularly when drugs such as ecstasy are used [4, 8, 14]. For example, during a 3-day EDM festival in New York City (NYC) in 2013, there were 22 drug-related emergency department (ED) visits, including two deaths [4]. With an approximately 40,000 attendees each day and a 85–90°F daily outdoor heat index, the NYC Department of Health and Mental Hygiene determined that these emergencies were due to heat exposure, crowding, and alcohol and drug consumption [4]. Further, a recent study of EDM nightclub and festival attendees in NYC estimated that one third (33.5) had experienced a drug-related adverse effect in the past year [7]. Due to a high likelihood of adverse events occurring during festivals, medical services must be well equipped and able to care for attendees who experience drug-related poisoning or overdose [5, 15].

Although extensive research has investigated prevalence of drug use at festivals, fewer studies have been conducted examining environmental factors within such events. Additional research identifying both festival- and person-specific risk factors is needed to examine how the festival environment may affect risky drug use and related adverse outcomes. To add nuance to this current gap in the literature, we conducted a qualitative study with key informants highly familiar with or experienced with use of new psychoactive substances (NPS) and festival scenes. We intend for results of this study to help identify modifiable risk factors and to inform prevention and harm reduction efforts in this high-risk scene.

## Methods

A purposive sample of 35 adult key informants was recruited through study flyers on social media and on drug information message board websites commonly frequented by psychonauts. Individuals were also via referral from other participants and recruited at harm reduction conferences. This loose design allowed for a variety of individuals throughout North America with different experiences to be interviewed. To be eligible, individuals must have (1) been age ≥ 18, (2) speak English, and report (1) being a drug checker, (2) a drug seller, or (3) report having extensive experience using or testing for new psychoactive substances (NPS). Drug checkers were defined as people who tested content of drugs for adulterants such as NPS (mainly at festivals) [16]. This included both those who volunteer at a drug checking organization and those who reported testing drugs casually for peers without such a formal affiliation. Drug sellers were people who reported selling or dealing illegal drugs or NPS to others. Extensive NPS use was not defined by a specific number of different compounds used, but the mean number of different NPS used was 6.9 (median = 5) with one participant reporting use of 24 NPS. This criterion was included as the parent study focused highly on NPS use and sales. A particular focus was on individuals experienced with drug use in nightlife and EDM festival scenes, with nightlife most commonly referring to late-night nightclub scenes. A screening was conducted over the phone or in person with the investigator to ensure eligibility. Interviews were conducted from 2015 through 2018. Those deemed eligible were either interviewed in person or interviewed over the phone after providing verbal consent to participate. Interviews were open-ended; however, some specific topics were discussed with all participants. Interviews were recorded and typically lasted about an hour. Participants were compensated $50 USD for interview completion.

Interviews were transcribed by a study staff member and multiple cycles of descriptive coding, subcoding, recoding, and pattern-coding of the transcripts were conducted [17] using Atlas.ti version 8 software [18]. Coding was conducted in an inductive manner in which codes progressively emerged rather than us choosing specific questions or codes a priori [19], and code saturation was reached within coding of the first 15 interviews, other than some later code definition changes and code-splitting [20]. A second reviewer double-coded transcripts for accuracy and the reviewers reached a high (95.5%) level of agreement. Dominant and/or repeated codes were then categorized into themes and quotations were extracted from transcripts that the reviewers believed to adequately summarize specific topics and themes. After a consensus was reached regarding classification of codes and themes, quotations in each domain were summarized to present a comprehensive picture. The New York University Langone Medical Center Institutional Review Board approved all study methods, and a Certificate of Confidentiality was also obtained from the National Institutes of Health to further protect participant confidentiality.

## Results

### Participant characteristics

As shown in Table 1, the majority of participants identified as male (71.4%) and white (88.6%), and the majority resided in the USA (91.4%). The average age was 26.7 (SD = 5.5, range 18–38), and the majority of informants interviewed identified as drug checkers (71.4%).

**Table 1:** Characteristics of key informants interviewed (*n* = 35)

	*n*	%
Age	*M* = 26.7	SD = 5.5
*Sex*		
Male	25	71.4
Female	10	28.6
*Race/ethnicity*		
White	31	88.6
Black	1	2.9
Hispanic	1	2.9
Other	2	5.7
*Country*		
USA	32	91.4
Canada	2	5.7
Mexico	1	2.9
*Informant category*		
Drug checker, user	18	51.4
Drug user only	8	22.9
Drug checker, seller, user	7	20.0
Drug seller, user	2	5.7
*Where recruited*		
Participant referral	13	37.1
Harm reduction or drug conference	9	25.7
Social media or study flyer	9	25.7
Investigator initiated Email	3	8.6
dance party	1	2.9

*M* = mean; SD = standard deviation. Ages ranged from 18 to 38 years

### Emergent themes

**Fig. 1:**
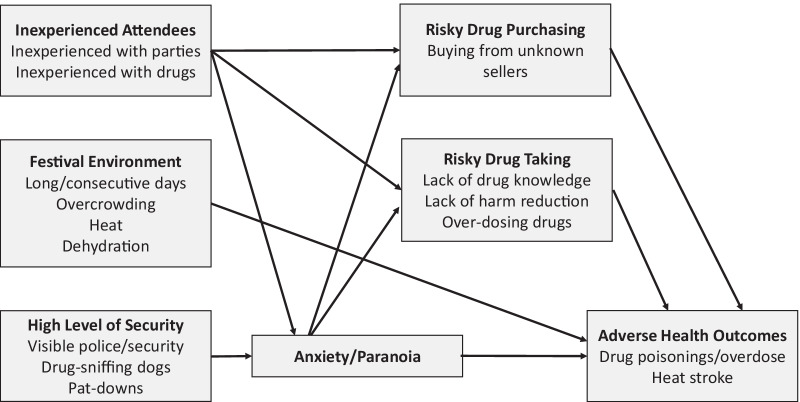
Theoretical model depicting directions of associations between themes

Four overall themes emerged: (1) inexperienced attendees, (2) risky drug purchasing, (3) risky drug use, and (4) environmental risk factors. Figure 1 presents a theoretical model presenting how these themes and subthemes appear to relate to one another. Specifically, we posit that inexperienced attendees and the festival environment (which includes various types of security) appear to increase risk for risky drug purchasing and risky drug taking, and in turn appear to increase risk for adverse health outcomes. Although anxiety and paranoia are not themes, they are discussed by participants, and we posit that they mediate many potential associations depicted in the figure.

### Inexperienced attendees

A substantial portion of festival attendees were viewed by informants as people with little to no experience with EDM party scenes or with party drugs. These inexperienced people appear to be more likely to be introduced to festivals as their first EDM events because festivals—especially larger festivals—tend to be more mainstream than underground nightclubs and raves. As such, these large gatherings tend to attract people who are not part of underground EDM scenes. Although nightclubs can also draw in wide audiences, festivals tend to draw in much wider audiences—geographically, and with respect to music preference and level of participation in the scene. Given the mass market appeal of these events, many people who do not frequent nightclubs attend, and as one informant pointed out, many of such attendees are not even EDM fans and simply attend for the experience.

It is this lack of familiarity with party scenes that appears to be a risk factor for risky drug purchasing and use because most of these attendees are assumed not to be seasoned party drug users.

People who come in from out-of-state, people that don’t have a regular club scene because they live in a small town—these are people that are driving 4, 8, 12 hours to get to a festival and they don’t do this every month. (Drug checker, male, age 32)

Festivals attract the novices. Festivals attract people that have never been there before, people that have never done drugs before. (Drug seller and drug checker, male, age 26)

Inexperienced partiers who attend festivals tend to be young and have less drug use experience than nightclub attendees, perhaps in part, because many nightclubs do not admit individuals younger than age 21. Such inexperienced attendees also appear to have fewer connections to people who can provide drugs, or to high-purity drugs. As pointed out by one informant, some attendees who live far away from a festival may indeed have access to trustworthy people who sell drugs near home, but they may feel too uncomfortable traveling to the festival while possessing the drugs. As such, these individuals may resort to purchasing drugs at the festival. This is discussed later in more detail as a risk factor for using adulterated drugs. Perhaps relatedly, these individuals who travel far to attend are also sometimes assumed to be under-informed about drug effects and harm reduction information. As one informant pointed out, “It’s not their fault they’re less aware or less practiced.” While some may assume that “experienced” drug use indicates frequent and perhaps even more problematic use, in festival contexts, lack of experience appears to be a risk factor for adverse drug-related effects.

Given that so many people at festivals use drugs like ecstasy to enhance their outing, it is likely that many inexperienced attendees assume that the festival is the best place to purchase their drugs. However, this is actually in sharp contrast to views held by more experienced attendees. For example, as one informant says, “Most real heads will buy their stuff beforehand or bring it into the festival.” Of course, this view is under the assumption that the new attendee even has a social network including people who can provide the desired drugs, but inexperienced attendees might simply not have the necessary social connections. On the other hand, clubbers—people who frequent nightclubs—were often described as being more experienced, and this in turn facilities connections with people who sell drugs (and perhaps more reliable or purer drugs). Without connections to more reliable sellers inside or outside of venues, inexperienced festival attendees may thus resort to risky drug purchasing activities. In particular, novices can be a target to be taken advantage of by “shady” drug dealers.

### Risky drug purchasing practices

Many informants described the purchasing of drugs inside festivals as risky. In this context, they typically referred to “risky” as referring to accessing low-quality products—often adulterated with drugs potentially even more dangerous than the drug they intend to consume. For example, many of our drug checker informants have frequently detected adulterants such as synthetic cathinones in ecstasy or Molly they tested for attendees.

Nightclub attendees are commonly assumed to have more reliable connections to drugs like ecstasy than non-attendees. This is because people who regularly attend nightclubs are often surrounded by people high on such drugs and such venues can also provide access to drugs. Even occasional attendees may have connections with other attendees or other locals who use or sell party drugs. As such, drugs can often be obtained from a somewhat trustworthy source or network both inside and outside of local nightclubs. But these connections are typically not just mere connections in which one can obtain drugs; these connections imply that the person providing the drugs is at least known to some degree. Rapport is also often part of these relationships which may imply a certain degree of trust. Indeed, when local clubgoers attend local festivals, they can likely utilize the same connections from club life, but those who do not attend nightclubs likely lack such connections. People who sell drugs at nightclubs vs. festivals can be very different. Below, some informants compare “dealers” between nightclubs and festivals.

You’re more likely to see the person again (in nightclubs) so they’ll want to actually have a reputation with you, more so than at a festival. So at least there is that semblance of reasonable interaction with someone procuring illicit substances. (Drug seller and drug checker, male, age 26)

The person you’re getting the drugs from (at a nightclub) would be a regular, a friend. You would see them again. There would be accountability if you acted like an asshole at a party. The biggest problem with festivals now is that there is no accountability. The person you buy drugs from at the festival on Day 1, you won’t even see on Day 2 or 3. (Drug checker, male, age 32)

Reputation and accountability refer to a seller who reliably distributes quality drug product. Multiple informants mentioned that festivals have a reputation for “fake” drugs being sold and that festivals are not trustworthy places to acquire quality drugs. For example, one drug checker observed that large EDM festivals tend to have a much lower percentage of high-purity drugs tested compared to other venues. Another drug checker further warned that adulterated drugs can be sold at both low and high prices, so price should not be an indicator of quality at such gatherings.

Multiple drug checkers pointed out that festivals often attract an “undesirable element”—people who are not EDM fans or part of the scene but rather attend to sell low-quality drugs. These are people willing to sell adulterated drugs and who have little to no interest in having return customers or repeat buyers. As one drug checker pointed out, repeat business is often not a concern if the festival contains 80,000 attendees he will never see again. This is typically not the case with trusted sellers or “house dealers” in nightclubs, who are well-known and widely trusted sellers who are sometimes thought to be sanctioned by nightclub staff. Both sellers and house dealers are known by many attendees and have a reputation for quality product to uphold in order to maintain a customer base.

Why is there any incentive for me to sell you anything real? You’re not going to see me the next day, and if you do, who cares. It’s a 3-day festival, you’ll be going back to your part of the country, they’ll be going back to theirs, that’s the end of it. (Drug checker and drug seller, male, age 27)

I think they’re more likely to get garbage at a festival because you don’t have the assumption of a repeat buyer. Like if I know my dealer, if I buy stuff from him and I have a shitty time on it, I’m not gonna buy his stuff again and he knows that. (Drug checker, male, age 30)

Adding to points made that buying drugs from strangers at festivals is risky, a few informants mentioned that such individuals can be easy to detect as they do not tend to provide a happy or peaceful vibe.

They’re clearly not part of the scene. They’re there just to sell drugs. They look associated with organized crime, like some gangs basically. They have sort of like prison tattoos and have the ‘don’t fuck with me’ stances…they’re not enjoying the music and not knowing anyone there. (Drug checker, male, age 30)

Those guys that are selling the caps (capsules containing Molly) just randomly at the festivals—you see them at every festival. It’s the same general group of guys. They look like they dress the same and you kind of know, those are the guys that are selling caps and all the little kids go to them. (Drug checker and seller, male, age 27)

Some informants compared this situation at festivals to more tight-knit scenes at nightclubs and warehouse parties and mentioned that attendees at smaller venues are more likely to know and look out for each other. Unknown sellers at such venues may be deemed suspicious and some venues are even known to remove “shady” dealers from the premises.

I’ve seen those kind of people (‘shady dealers’) kicked out. Everyone’s got each other’s back or watching out for each other. It’s not just some random person you’re getting stuff from. It’s someone that you know and rely on. (Drug user only, female, age 34)

However, it should be noted that a few informants mentioned that they are in fact aware of drug sellers at festivals who do quality control their product. One also mentioned a constellation of house dealers (sellers familiar but not necessarily sanctioned like the original definition of “house dealer”) around some small festivals, but not around very large events. As such, it was noted that some festival drug dealers do care about customers’ well-being and some genuinely want to help enhance the experience for patrons. Although as one informant pointed out, similar to known sellers at nightclubs, quality product is essential in order to maintain clientele trust.

Because of the fact that you’re gonna see these dealers at multiple events, it’s much more likely that these dealers are gonna quality control the product just because they don’t need to get yelled at by 500 people. (Drug checker, male, age 32)

Given that more experienced drug users are more likely to understand the risk of using adulterated product, some may assume that people who purchase drugs as festivals from strangers are not aware that the drug can be bunk. However, while many are in fact unaware, many are aware and are simply willing to take a risk in order to enhance their time at the event. Feelings of anxiety and desperation in particular appear to prompt some attendees to simply purchase drugs from “shady” people or from the first person offering to sell—especially if hours have been spent trying to find a seller.

A lot of problems with first timers or people that don’t really know better is they just won’t know where to look and the second someone offers something to them, they’ll be like oh, great, I was just looking for this. (Drug checker and drug seller, female, age 19)

A major problem with this, as a drug seller informant points out, is that all it takes is for one dealer to distribute 100 pills or baggies containing dangerous drug product to lead to 100 intensive care situations at the festival. But risky drug purchasing is only one particular risk factor for attendees having dangerous drug exposures. Risky drug *use* at festivals was another risk factor commonly discussed by informants.

### Risky drug use practices

Compared to other venues such as nightclubs, some informants also discussed drug use behavior as tending to be riskier at festivals. Much of this appears to relate to inexperience with drugs and party scenes and lack of social connections (as previously described), but *also* to lack of drug education from people who use. For example, a few drug checkers pointed out that many people who attend festivals take drugs like Molly and have no idea what to expect. As such, people who use may not only not have knowledge about drug effects to expect, but they also likely do not know how to reduce potential harm that can result from use in such an atmosphere.

Festivals attract people that don’t know when to say no, that don’t know when their body is telling them bad signs, that don’t know when to say I need help. (Drug seller and drug checker, male, age 26)

The issues this informant points out can be exacerbated by the fact that a lot of attendees go into “vacation mode” at festivals, meaning that their day or weekend at the festival equates to an expensive trip or getaway with an aim to maximize pleasure. This leads a lot of attendees to “go hard” and to party harder than they would in other environments such as nightclubs.

I think there’s a cultural acceptance of being able to get really, really fucked up at a festival and go crazy. They really want to escape. It’s their vacation and they’re going to party as hard as they can. Whereas a raver or [person attending a] nightclub, you have work on Monday. (Drug checker, male, age 30)

Further, while many inexperienced users at least attempt to be careful regarding their use at festivals, there are some young people are not very mindful at all about what they ingest and may aim to become “hilariously intoxicated.”

There’s this impetus to really kind of push the envelope. You know there was this story of kids in Australia that got a festival shut down because a bunch of 19-year-olds were playing a game of who could take the most drugs. (Drug checker, male, age 32)

It should be noted, though, that while over-use of drugs at festivals does appear to be common, in many cases it is actually due to attendee desperation. This is discussed in more detail in the *security* subsection below, but briefly, many attendees do not carefully administer their drug doses inside festivals in effort to not be seen, and others take large doses on the way into the festival to avoid being caught by security.

Given that festivals are large annual events that are often compared to mini-vacations, even seasoned drug users can go overboard regarding dosing and re-dosing, combining drugs, and not following basic harm reduction recommendations like adequate rest and hydration. All of this can place people at risk for adverse drug-related outcomes. Much of this is related to the attendee’s mindset, but as we discuss in the next section, risky drug use and adverse health outcomes also relate directly to festival environment conditions.

### Festival environment

Study informants discussed various environmental factors somewhat unique to festivals that can increase risk of drug-related adverse effects. In fact, these conditions can also lead to adverse health consequences even in the absence of drug use. We summarize the factors discussed as follows: crowding, heat and dehydration, long/consecutive event days, medical response to emergencies, and security and police officer presence.

#### Crowding

Indeed, nightclubs can be crowded—to or beyond legal capacity—but crowding at festivals can be a much different phenomenon. First, the sheer size of the crowd can be overwhelming as many festivals contain hundreds of thousands of attendees on the same day. Second, at festivals, attendees tend to form a dense crowd facing DJs as they perform. These crowds appear to be denser than typical nightclub crowds in which even crowded dancefloors still tend to have some space between attendees. As pointed out by a few informants, being in a trapped in crowd can be anxiety-provoking due to a sense of lack of control, especially when on a drug like ecstasy or LSD.

Crowding, overwhelming stimuli such as bright light shows and loud music, when combined with drugs can be particularly anxiety-provoking, especially if someone is not experienced with the drug used or not familiar with this type of environment.

It’s a recipe for bad times. You have people that have never done drugs before and they’re like I’m gonna go in a large crowd of people with music in a setting I can’t control at all and do this drug that’s gonna be psychoactive. (Drug checker, male, age 30)

Crowding can also make it easy to be displaced from your friends, and it can make it difficult to navigate, to acquire food or water, and it can be difficult to seek and receive medical assistance if needed. As such, crowding can also increase the likelihood or effects of potentially serious issues such as overheating and dehydration.

#### Heat and dehydration

Although festivals are held in all sorts of climates, many are held during summer months, and some are held in desert settings which can be very hot. For example, in such settings, the temperature can easily remain over 90°F throughout most of the day and dancing in the sunlight can make it feel even hotter. The heat alone can be exhausting, even without drug use and extensive dancing. Such conditions can be even more exhausting for attendees not acclimated to such conditions. Given that MDMA in particular can affect regulation of body temperature, this drug in particular appears to have the potential to exacerbate potential adverse effects caused by outside temperature. As an informant points out, hospitalizations and deaths related to ecstasy use are commonly blamed solely on use of ecstasy/MDMA without people considering environmental effects.

So, we had one festival where they attributed deaths to ‘bath salts’ or Molly. [These] were actually just heat-related deaths and they weren’t [from] drugs. (Drug checker, male, age 30)

Related to the issue of overheating and possible heat stroke, it is easy for partiers to become dehydrated at such festivals, even without extreme heat conditions. Smaller venues like nightclubs seem to have more consistent access to water—whether it is free (from fountains or sinks) or available for a fee (through bottles). Indeed, bottled water tends to be expensive (e.g., $5 USD) at various types of party venues which can discourage some people from spending extra money to stay hydrated, but regardless of price, water can be more difficult to obtain at a large festival. There tends to be long lines to purchase water or to obtain free water from a fountain. Waiting for 15 min in the heat for water can be exhausting and some attendees choose not to go wait in line for water in order not to lose their space in front of the stage. As such, some attendees chose to remain dehydrated rather than miss a close-up view of performers.

While lack of water can obviously lead to dehydration quickly, alcohol and drug use tend to further lead to dehydration, which is further compounded by the heat. Not only do many dehydrated people drink alcohol at festivals, but one informant mentioned that alcohol can actually be easier and cheaper to obtain than water at some festivals. This can encourage some people to drink alcohol instead of water. With respect to drug use, psychostimulants in particular can cause dehydration. Therefore, using a drug like ecstasy/MDMA can be dehydrating within itself, but this combined with lack of water and/or use of alcohol in such hot conditions can lead to dangerous consequences. This informant claims to have almost lost his life from such a situation.

Alcohol really dehydrates you and if you’re taking a stimulant or something that’s going to really dehydrate you, and you don’t have enough water, that’s a problem. That’s a huge issue at festivals. People die a lot from dehydration. I almost did. It was pretty bad one time. (Drug user only, male, age 21)

The environmental conditions discussed thus far all appear to increase risk for adverse health effects, particularly among people high on psychostimulants. However, as we discuss below, this situation being extended for many hours or possibly multiple days in a row can be particularly dangerous.

#### Long, consecutive days

Extreme environmental conditions combined with drug use and dancing within a single day can be risky, but according to informants, risk is increased by the length of festival days and by consecutive festival days. Many festivals are open from roughly noon to midnight—an extensive time period for an individual to be active in a party atmosphere. Most major festivals last two or three consecutive days; for example, Friday, Saturday, and Sunday. This was pointed out as a risk factor for adverse health effects.

Partying for multiple days at a festival can lead to a 3-day cycle of drug use, dehydration, malnutrition, and exhaustion. Even without drug use, such festival environment conditions can be exhausting, but drug use does appear to exacerbate these other conditions, and drug and alcohol use can also lead to poor sleep and hangovers. As such, effects from behavior on the previous day can easily spill into the next day.

If you go on a three-day binge, your body is gonna be hurting. People are not sleeping properly, not eating properly; they’re in the sun all day. (Drug checker, female, age 28)

They’re also doing them (drugs) longer at multi-day festivals, so you start to pile on extreme polydrug use and sleep deprivation and alcohol and that’s when things start to get weird. (Drug checker, male, age 30)

Being high on a drug for up to 12 h at a festival can be exhausting, and this is aside from potential pre-gaming and post-festival use. Repeating this pattern on multiple days—especially among people who are less used to such conditions—can lead to serious exhaustion, especially given other festival conditions. As one informant points out, drug use in such extreme environments multiple days in a row appears to increase risk of an adverse effect occurring.

It’s not that the drug was so toxic that they took it on Day 1 and then collapsed. Usually, it’s because they’ve become dehydrated and now they’re doing it again, and this is compounding. So, by the time it’s the second or third day, their body is not handling it well. (Drug checker, female, age 28)

Given potential widespread drug use, exhaustion, dehydration, and overheating, it should not be surprising that festivals tend to have hundreds of medical emergencies. However, medical response can be limited, and when medical emergencies are not attended to on time or in a sufficient manner then increased risk of morbidity and even mortality can occur.

#### Medical response to emergencies

Not only are people at risk for medical emergencies at large festivals due to a variety of factors previously discussed, but people experiencing an emergency may actually be hesitant to seek help. This is because they may fear getting in trouble or because they might not want to leave the festival. As noted earlier, many people who attend festivals are in “vacation mode.” Many attendees plan their weekend for months, and when they are there and experiencing an adverse event, they may choose to sit to the side to see if they feel better, rather than seeking help. On an average club night, it is likely that not feeling well will lead the attendee to simply take an Uber home or to a hospital as necessary, but when so much time and money have been invested, the attendee may simply try to fight off the illness. This can be viewed as a lack of self-response to possible emergencies.

For those who choose to seek medical help, they then need to be able to locate the proper staff or medical tent. If someone is experiencing an adverse event in main festival areas that are crowded, it may go unnoticed by festival staff, especially if no one reports the incident. Major adverse events can occur anywhere at a festival. In fact, as one informant pointed out, there was a well-publicized death of an attendee waiting on a long line to catch an Uber to leave a festival.

Another issue regarding medical response is that high-quality medical care may not be available for severe drug-related emergencies. One informant pointed out that some medical personnel at festivals have little to no experience treating people experiencing drug poisonings. Lack of proper equipment to treat major medical emergencies may be an even a bigger limitation; however, in many cases, attendees can be transported to a hospital via an ambulance. Yet, a major issue for some festivals is that they are far away from hospitals. This can even be the case in urban areas as large fields that hold festivals are often removed from denser areas with hospitals.

Most music festivals are removed substantially from any type of city area with a hospital. You’re going to have a better chance of surviving if you’re in a nightclub because an ambulance can come right away, there’s people around you, there’s security. There’s not a lot of places you can go where you are unseen and can die in the corner without anyone noticing. But in a music festival, you could wander off. A lot of times these are in the middle of the woods, you can get lost, and you can have a terrible experience without anyone noticing you. There’s medical onsite but if it’s a major emergency and the next hospital is four hours away, you have less resources available if you do have a negative reaction. (Drug checker, female, age 29)

The ability to receive quality medical care may be limited, but to some, seeking medical care is seen as a way to incriminate oneself for drug use or possession. As such, perceived consequences for seeking care can range from getting removed from the festival to being arrested for possessing a drug.

You’re much less likely to seek help immediately, you’re much less likely to start dealing with a problem seriously because you’re terrified of the NYPD or whichever law enforcement you’re dealing with. (Drug checker, male, age 32)

You have to tell the crowd that you can go to the medical tent and cops won’t be called because there’s this fear where if you’re having a bad time or you think somebody’s in trouble, if you reach out for help, everyone’s gonna get busted. And that keeps people from going for help when a situation could be fixed. (Drug checker, male, age 30)

The informant further mentioned that one festival he worked at had a medical tent with a sign saying, “We won’t tell the cops anything. Everything here is confidential.” However, they were supposedly asked to remove the sign by security. Similar to Good Samaritan laws in which people who possess drugs that call for medical help from home are not prosecuted, informants argue that this is needed for attendees to be willing to visit medical tents in the case of a drug-related emergency. Fear of law enforcers is seen as a major fear for attendees who experience a drug-related emergency at festivals.

#### Security and police officers

Security and police officers can hinder attendees from seeking medical help when needed, but as reported by various informants, security and police presence is also associated with anxiety and paranoia among attendees. Similar to nightclubs, there is a variation with some festivals having more laxed security and others having more intense security. It appears to be high-level security in particular that can make both drug users and nonusers alike feel uncomfortable because they feel like they are being observed. This can start with pat downs at the entrance, sometimes followed by multiple checkpoints, and then visible security inside. Security at festivals—particularly when mass-hired for large events—may also not be experienced overseeing crowds of people on drugs, and they may be overly hostile or intimidating. The potential for undercover officers observing attendee behavior is another threat and reason for many attendees to be paranoid. Further, as one informant pointed out, one festival was even rumored to have cameras able to read text messages from far away. This information, even if exaggerated, is enough to feed attendee paranoia.

I think it (police presence) also creates a level of anxiety at the event. Police presence is not something that anyone that’s fucked up wants to see even if they’re having a great time. If they see a cop pushing somebody to the ground and arresting them, that’s hugely destabilizing (Drug checker, male, age 32)

Aside from overall anxiety related to seeing police or security presence, this can increase risky drug-taking (and risky drug purchasing) behavior. Attendees who purchase drugs inside the festival may resort to purchasing from the first stranger who offers—simply to get the task out of the way. However, without cautiously choosing which seller to buy from, bunk product may be purchased. With respect to use, as one informant points out, security presence can “generate lack of self-care and lack of harm reduction.” As such, attendees may not measure doses they take or take too much of a drug abruptly in order to get it over with and not get caught. For example, someone may quickly take a large dose in the middle of a crowd, or the attendee may take a large dose in a port-o-potty and hope the dose is large enough to not have to return to re-dose for some time. This can be dangerous as this increases risk for overdose, and this is likely more common during daytime hours when it can be more difficult to use out in the open and not be seen.

Using and purchasing drugs inside of festivals can be risky, but informants pointed out that drug-taking behavior before or while entering festivals can be particularly risky in response to fear of getting caught with drugs. Many festivals have intense security pat downs and bag checks at the entrance, and police may also be nearby to arrest people caught with drugs. Some festivals also have drug-sniffing dogs accompanied with officers which adds yet another level of anxiety for many people trying to sneak their drugs in. While many people find security checks laughable and sneak in drugs without much paranoia, many attendees panic and pre-dose while approaching the festival. Pre-dosing within itself does not appear more dangerous than dosing inside the festival, but the risk is that many people take the full batch of their drug on the way in attempt to get high and not get caught. This can lead to serious adverse effects.

They’re not gonna be able to get the drugs in so they just take everything on line. They don’t know how long the line is gonna take and generally those lines don’t have access to water. I think it was Electric Daisy Carnival they had people passing out on the security line from heat before they even got into the concert. (Drug checker, male, age 30)

I’ve seen people collapse in line. I’ve seen people go out in stretchers 10 minutes within doors opening. People are just gonna end up doing it more recklessly because they’re paranoid and freaked out by getting caught. Once it’s already in your body, no one is arresting you really. If they find something on you, that’s when you get in trouble.” (Drug checker, female, age 28)

In the following case, an informant claims to have ingested 800 mg of MDMA in a panic after learning about a security check on his way into a festival. Whether or not such a large dose was indeed ingested, this is a very dangerous behavior—all in attempt to avoid being turned down at the gate or to avoid arrest.

[I] accidentally took 800 mg of MDMA because my friends were like, ‘Holy shit, they’re going to check us and we won’t be able to get in. We have to take whatever we have right now.’ I didn’t really know what to do at that point and I was doing the calculations in my head. I’ve done almost an entire gram of MDMA and that was really intense. (Drug checker, male, age 23)

So, while security and police presence are in fact much needed at festivals, this presence appears to increase the risk for anxiety and risky drug purchasing and use.

## Discussion

Dance festivals tend to be high-risk events for drug-related adverse outcomes and more research was needed to determine which festival-specific risk factors in particular make attendance risky. We identified four main themes focusing on festival attendance as a risk factor for risky drug use and related outcomes: attendees being inexperienced, risky drug purchasing practices, risky drug use practices, and festival environment as a risk factor.

A major theme was that festivals often attract individuals with little to no experience with EDM parties or associated drug use. This inexperience appears to be linked to risky drug purchasing and use at festivals. We found that more inexperienced drug-using individuals tend to purchase their drugs from unknown and untrusted sellers. Nightclub attendees, however, were viewed as being more experienced in purchasing and using party drugs. These findings add to previous literature, suggesting that it is more ideal to purchase from a known or trusted seller to acquire more consistent drug product and to minimize potential adverse health risks associated with adulterated drugs [21]. Research has indicated that interpersonal relationships based on trust among drug sellers and people who use is an important factor for safer drug use [22]. Inexperienced users with no such connection to trustworthy sellers often appear to assume that festivals are the ideal place to purchase drugs. However, drugs purchased from unknown sellers, particularly at festivals, can lead to purchasing of adulterated drugs and therefore poisonings [23]. Given that inexperience appears to be an issue, education, prevention, and harm reduction efforts should likely be aimed toward such individuals in particular. Specifically, education is needed to alert new attendees in particular about the potential dangers of purchasing drugs from strangers at festivals.

According to our informants, drug use at festivals tends to be riskier compared to other party venues. We found that many people are likely to go into a “vacation mode” at festivals, and over-use drugs is common to maximize pleasure. Even experienced attendees can easily go overboard regarding dosing and consuming drugs too frequently within a short period of time in the given atmosphere. This finding is in consistency with studies reporting that consumption of particular party drugs is often done in celebratory contexts. For example, a meta-analysis reported that escapism and excitement are motivations for drug use at festivals [24]. Similarly, in wastewater analysis conducted in Australia, it was demonstrated that use of drugs, especially cocaine and MDMA, significantly increased during the peak holiday period [25]. Therefore, given the high prevalence of drug use at festivals, exacerbated risk of drug-taking due to attendees’ mindset and lack of care in administering drug doses can increase risk for adverse drug-related outcomes.

Festival-specific factors such as visible security presence can hinder harm reduction practices against risky drug use, even among experienced attendees. We found that presence of high level of anxiety-producing security can lead to purchasing drugs inside the festival and reduced ability of controlled dosing both before entering and inside the event. These findings support previous studies. For example, at a festival in Australia, 39% of attendees who expected a drug-sniffing dog near the entrance reported obtaining drugs inside the festival grounds instead of buying them beforehand [26]. Another study conducted at an EDM festival in Melbourne reported that buying drugs at festivals hindered attendees’ ability to use common harm reduction techniques such as testing the drugs [27]. Similarly, and as previously mentioned, many attendees are likely to not carefully administer their drug doses inside festivals. Interviews suggest that anxiety and paranoia associated with security at festivals can lead people to not carefully administer drug doses and this can lead others take large doses on the way into the festival to avoid being caught by security. In previous studies, it has been shown that some attendees pre-load drugs before entering an event due to fear from security [26, 28] and experience overdose [29]. These findings can inform future studies to better determine the role of security as a festival-specific risk factor and should call into question the efficacy of policing of drug use at festivals.

In addition to visible security presence, risky drug use practices and adverse health outcomes directly relate to other festival environment conditions. The interviews revealed that several festival-specific environmental factors such as crowding, heat and dehydration, long and consecutive days of partying, and lack of sufficient medical equipment can increase the risk of adverse health outcomes. Our respondents informed us that being in a crowd during a large festival can be anxiety-producing, especially if someone is on a drug. Crowding, in combination with other factors such as repeated drug use while exposed to heat over long and consecutive days, appears to increase risk for adverse health effects. These findings further corroborate studies in regard to festival-specific environmental factors and adverse health outcomes. For example, in a case report focusing on twelve festival attendees who were admitted to an ED, the authors reported three cases of death due to prolonged outdoor physical exertion by long hours of dancing and drug use, which resulted in heat stroke [14]. In another study of a large music festival, it was shown that heat-related patient presentations increased with average temperature [30]. Lack of rest/sleep (and eating) due to multiple days of attending also appears to increase risk of adverse outcomes, and those who travel to festivals outside of their home area are also at high risk for adverse drug outcomes [4, 31, 32]. Given these cases, adequate medical response to emergencies at festivals is critical. It has been shown that medical tents at festivals are often efficient and reduce the need for ambulance transports [33]. However, as many of our informants pointed out, self-response to possible emergencies may be low at festivals due to fear associated with incriminating oneself for drug use or possession. Sufficient medical care may not be available for severe drug-related emergencies, which is consistent with events in festivals in which medical personnel are not fully equipped [34] or not knowledgeable enough [35] to deal with drug-related incidents.

We believe these qualitative results can inform quantitative research to better determine the actual risk associated with each theme and subtheme discovered. However, even despite the lack of quantitative research on this topic, we believe many of the risk factors that arose in themes may in fact be modifiable. While we believe that experience is the only true remedy for inexperience, we do believe education can target those who are most inexperienced. Festival environments can be difficult to modify assuming promoters do not have full control over when the festival can be held, but times of year when it is hottest may be able to be avoided. Further, stricter capacity limits can help limit overcrowding. Regardless, adequate water supply is needed as well as adequate areas for patrons to rest and cool down, especially when it is hot outside. Security may be a more complicated factor given that festival promoters likely need to show that they are trying their hardest to keep drugs out but also limit the risk of attendees engaging in riskier drug use. Perhaps confiscating drugs rather than arresting those caught possessing them could lower anxiety, although then the deterrent effect of security may then diminish. Even though laws did not emerge as a theme, it is important to consider that such macro-level factors are important regarding the health of attendees. The Illicit Drug Anti-Proliferation Act (commonly referred to as the RAVE Act) has been a major deterrent against party promoters disseminating drug education and harm reduction information at venues in the USA, and from allowing drug checking to be conducted on the premises [16]. We believe that finding ways to modify existing laws and environmental risk factors in particular could have the greatest impact regarding reduction of drug-related risk behavior and adverse drug-related outcomes at festivals.

This study is not without limitations. This study was exploratory in nature and the sample size was relatively small; however, we believe that a smaller sample size suffices given that we consider participants key informants or experts in the topic of interest. All participants reside in North America which can be a limitation as we do not know whether or to what extent they are experienced or knowledgeable with respect to festival culture in other continents. Local and national policies can differ across countries, and this can lead to different behaviors. For example, whether or not drug education and services like drug checking are legal or condoned by other governments, festivals in some other countries have drug checking programs located inside festivals to provide harm reduction [36, 37]. In the USA, this rarely occurs. As such, attendees in the USA may be more likely to lack education about drug use and possible drug contents compared to festivals in some other countries. Relatedly, we must keep in mind that dance culture varies across countries, so experiences of attendees may be vastly different throughout the world.

## Conclusion

This study determined several modifiable festival-specific risk factors which can increase the risk of drug-related adverse outcomes at festivals. Festival-specific factors such as heat, crowding, and visible security, combined with attendance of individuals who are inexperienced in this scene can lead to risky drug purchasing and risky drug use practices. Results of this study can both inform future research and inform future prevention and harm reduction efforts in this scene.

## Data Availability

Not applicable.
